# Impact of Hepatic Steatosis on the Antiviral Effects of PEG-IFN*α*-2a in Patients with Chronic Hepatitis B and the Associated Mechanism

**DOI:** 10.1155/2020/1794769

**Published:** 2020-06-26

**Authors:** Huiqing Liang, Yaoyu Liu, Xiaoqian Jiang, Xiaoting Zheng, Jinmo Tang, Jiaen Yang, Hongli Zhuang, Penghua Lai, Li Peng, Zhenying Guo, Shanshan Cai, Dan Luo, Lingxia Xu, Qianguo Mao, Shaodong Chen

**Affiliations:** ^1^Hepatology Unit, Xiamen Hospital of Traditional Chinese Medicine, Xiamen, Fujian, China; ^2^Department of Traditional Chinese Medicine, Medical College of Xiamen University, Xiamen, Fujian, China; ^3^Fujian University of Traditional Chinese Medicine, Fujian, China; ^4^Department of Traditional Chinese Medicine, No.1 Hospital Affiliated to Xiamen University, Xiamen, Fujian, China

## Abstract

**Objective:**

To investigate the risk factors for hepatic steatosis in chronic hepatitis B (CHB), to determine its correlation with liver necroinflammation and fibrosis and response to peginterferon alpha-2a (PEG-IFN*α*-2a) antiviral therapy, and to explore the mechanisms underlying the poor antiviral effect of PEG-IFN*α*-2a in CHB patients with hepatic steatosis.

**Methods:**

We analysed the impact of hepatic steatosis on the antiviral effect of PEG-IFN*α*-2a on CHB patients in a cohort of 226 patients who underwent pretherapeutic liver biopsy. To assess the complete response (CR), virological response (VR), and biochemical response (BR), the 226 patients were treated with PEG-IFN*α*-2a for 48 weeks and were followed-up for 24 weeks. The expressions of hepatitis B surface antigen (HBsAg) and hepatitis B core antigen (HBcAg) in the liver tissue were detected in all patients to explore the possible mechanism of hepatic steatosis with regard to antiviral effects.

**Results:**

The patients were divided into four groups based on the severity of hepatic steatosis: 119 with no steatosis, 76 with mild steatosis, 22 with moderate steatosis, and 9 with severe steatosis. In the hepatic steatosis groups, the proportions of male patients, patients aged >40 years, patients with hyperuricaemia, patients with a BMI > 23 kg/m^2^, and total cholesterol (TC), triglyceride (TG), glucose (GLU), and uric acid (UA) levels were significantly higher than those in the group without steatosis, whereas the alanine aminotransferase (ALT) and aspartate transaminase (AST) levels were significantly lower than those in the group without steatosis. The multivariate analysis results indicated that a BMI > 23 kg/m^2^ was independently associated with CHB patients with hepatic steatosis; the levels of baseline AST and UA were independently associated with CHB patients with significant hepatic steatosis, and the baseline AST level was independently associated with significant liver fibrosis. After 48 weeks of treatment and 24 weeks of follow-up, the rates of CR, VR, and BR had gradually decreased, whereas the severity of hepatic steatosis had increased.

**Conclusion:**

Hepatic steatosis can reduce the efficacy of PEG-IFN*α*-2a in the treatment of CHB patients, and its mechanism may be related to the different HBcAg expression patterns in liver tissue.

## 1. Introduction

Chronic hepatitis B (CHB) and nonalcoholic fatty liver disease (NAFLD) are two major chronic liver diseases in China. As lifestyle and dietary structure have changed, the number of CHB patients with hepatic steatosis has increased. The incidence varies widely around the world, fluctuating between 27 and 51% [1, 2], with a median prevalence of 28%. A report of 1915 liver biopsy cases in China revealed that the incidence of hepatic steatosis in CHB patients was 14% [3]. Whether CHB with hepatic steatosis has an aggravating effect on the progression of liver disease is unknown. A study in which liver biopsies were performed in 64 CHB patients with hepatic steatosis by foreign scholars revealed that 86% of patients had varying degrees of fibrosis [4]. Among them, 39% had progressive hepatic fibrosis, but no control group was established. In a study of 164 CHB patients, Altlparmak et al. [5] compared patients with and without hepatic steatosis and suggested that there was no significant difference between the two groups in inflammation and fibrosis scores. Another study by Chen and Shi [6] reported that the degree of hepatic steatosis in CHB patients was negatively correlated with liver inflammation and fibrosis. Data are scarce regarding the impact of hepatic steatosis on antiviral treatments, particularly in terms of peginterferon alpha-2a (PEG-IFN*α*-2a) treatment for CHB. A few earlier studies had revealed no significant association of hepatic steatosis with the antiviral effect in CHB patients [7, 8]. However, some new studies have shown that hepatic steatosis may reduce the antiviral efficacy of treatments for CHB patients [9–11]. Future studies examining the potential relationship between hepatic steatosis and the antiviral effect in CHB patients are necessary. This prospective cohort study assessed four different degrees of hepatic steatosis in CHB patients to investigate the effects of hepatic steatosis on CHB pathology and whether hepatic steatosis affects the efficacy of PEG-IFN*α*-2a. Therefore, this study was conducted to compare the expression patterns of hepatitis B surface antigen (HBsAg) and hepatitis B core antigen (HBcAg) in hepatocytes in different groups and to explore the possible mechanism by which hepatic steatosis affects the efficacy of PEG-IFN*α*-2a.

## 2. Materials and Methods

### 2.1. Patients

This prospective cohort study included 245 naive CHB patients who underwent percutaneous liver biopsy from May 2010 to 2016 at the Xiamen Hospital of Traditional Chinese Medicine. All patients underwent percutaneous liver biopsy, and their hepatic tissues were confirmed to exhibit or not exhibit steatosis by liver histological pathological examination. Patients were treated with PEG-IFN*α*-2a for 48 weeks and followed up for 24 weeks. Six patients were excluded due to abnormal thyroid function, eight patients were excluded due to an absolute neutrophil count in the peripheral blood ≤0.5 × 10^9^/L, and 5 patients were lost to follow-up due to emigration. The remaining 226 patients formed the study cohort. The patients were divided into four groups based on the severity of hepatic steatosis: 119 had no steatosis (group S0), 76 had mild steatosis (group S1), 22 had moderate steatosis (group S2), and 9 had severe steatosis (group S3). All patients were defined as HBsAg- and HBcAg-positive for at least 6 months, with baseline HBV DNA levels greater than 20,000 IU/mL, ALT levels elevated between 2 and 10 times the upper limit of normal (ULN), and hepatic histopathology results demonstrating steatosis. Patients with a history of alcohol intake >140 g/week, concomitant aetiologies including chronic hepatitis C or D coinfection or superinfection, autoimmune hepatitis, drug-induced liver injury, HIV coinfection, or evidence of immune suppression were excluded. The study protocol was approved by the Ethics Committee of the Xiamen Hospital of Traditional Chinese Medicine. All patients provided informed consent. Baseline pretreatment evaluations included collecting data on age, sex, course of disease, height, and weight, liver inflammation activity, fibrosis stage [12]; measurement of the HBV DNA levels; detection of HBV genotype; and quantification of the HBV e antigen (HBeAg), HBsAg, ALT, AST, CHO, TG, GLU, and UA levels. All data were assessed in the central laboratory of the Xiamen Hospital of Traditional Chinese Medicine. The HBV DNA load was assessed by fluorescence quantitative PCR with a low detection limit of 500 IU/mL using the COBAS TaqMan HBV Test (Roche's Lightcycler fluorescence quantitative PCR system, Shanghai, China). Quantification of the HBsAg, HBsAb, HBeAg, and HBeAb levels was performed by an electrochemiluminescence immunoassay using a Roche Elecsys 2010 system (German Roche Diagnostics Ltd, Germany). HBV genotypes were assessed by the PCR reverse dot blot hybridization method (Shenzhen Yaneng Biotechnology Co., Ltd).

### 2.2. Liver Biopsy and Histology

All patients provided informed consent for the liver biopsy procedure. The liver biopsy was performed using 16G biopsy needles guided by ultrasonography. A qualified biopsy specimen was either a minimum of 1.5 cm long or displayed for 6 or more portal tracts. The specimens were fixed, paraffin-embedded, and stained with haematoxylin and eosin (HE staining). Scheuer's scoring system was used to semiquantify the histological necroinflammation from G0 to G4 and the fibrosis stages from F0 to F4 by two pathologists independently who were blinded to the patients' biochemical and virologic results. Fibrosis was evaluated in all specimens by subjecting them to Masson Trichrome staining. Biopsied tissue sections were cut into 4 mm samples from prepared representative tissue blocks (lumps) and then placed in a paraffin oven to remove most of the paraffin. To complete deparaffinization, the specimens were then passed through xylene and a series of alcohol dilutions for 5 minutes and underwent microwave treatment using antigen retrieval for 10 minutes. After soaking in the methanol solution with 3% H_2_O_2_ for 5 minutes to block endogenous peroxidase, the peroxidase-conjugated Envision Kit (Envision-PO, Envision System; DAKO, Carpinteria, CA, USA) for rabbit primary antibodies was applied to the specimens for immunohistochemical staining of HBcAg in the hepatocytes.

**Figure 1:**
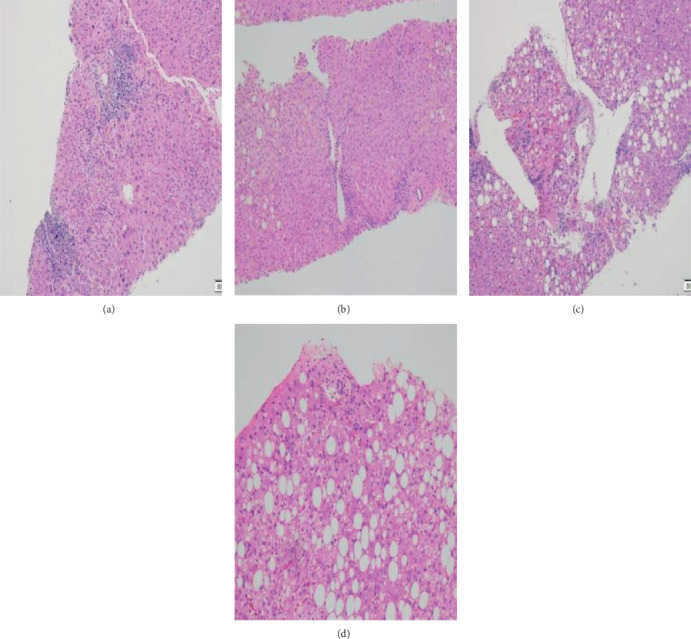
The distribution of hepatic steatosis was categorized into four types in this study. (a) In the S0 group, less than 5% of hepatocytes were affected by steatosis (HE staining, ×200). (b) In the S1 group, 5–33% of hepatocytes were affected by steatosis (HE staining, ×200). (c) In the S2 group, 34–66% of hepatocytes were affected by steatosis (HE staining, ×200). (d) In the S3 group, more than 66% of hepatocytes were affected by steatosis (HE staining, ×200).

Hepatic steatosis was categorized as follows: no steatosis (steatosis affected less than 5% of hepatocytes, S0), mild steatosis (steatosis affected 5–33% of hepatocytes, S1), moderate steatosis (steatosis affected 34–66% of hepatocytes, S2), and severe steatosis (steatosis affected more than 66% of hepatocytes, S3) (Figure 1) [13].

HBcAg expression patterns were classified as follows: no HBcAg expression (HBcAg-negative), cytoplasmic expression (cHBcAg), cytoplasmic dominant expression (cdHBcAg), and cytoplasmic and nuclear mean expression (mHBcAg).

**Figure 2:**
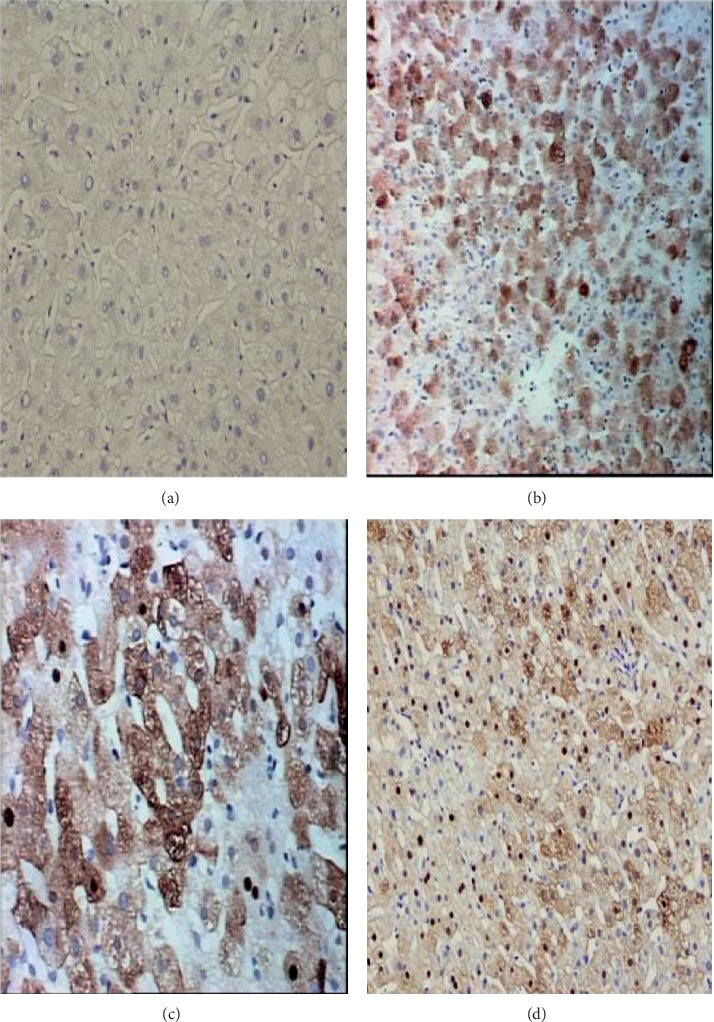
The distribution of HBcAg in the hepatocytes of CHB patients was classified into four types. (a) The HBcAg-negative type showed no HBcAg expression in either the nucleus or cytoplasm of the hepatocytes (immunohistochemical stain for HBcAg, ×200). (b) The cHBcAg type showed HBcAg expression only in the cytoplasm but not in the nucleus of hepatocytes (immunohistochemical stain for HBcAg, ×200). (c) The cdHBcAg type showed more than 2/3 HBcAg expression in the cytoplasm of hepatocytes (immunohistochemical stain for HBcAg, ×200). (d) The mHBcAg type showed 50% HBcAg expression in the nucleus and 50% HBcAg expression in the cytoplasm of the hepatocytes (immunohistochemical stain for HBcAg, ×200).

The HBcAg-negative type showed no HBcAg expression in the nucleus or cytoplasm of the hepatocytes, the cHBcAg type showed HBcAg expression only in the cytoplasm but not in the nucleus of the hepatocytes, the cdHBcAg type showed more than 2/3 of HBcAg expression in the cytoplasm, and the mHBcAg type showed 50% HBcAg expression in the nucleus and 50% in the cytoplasm of the hepatocytes (Figure 2) [14].

**Figure 3:**
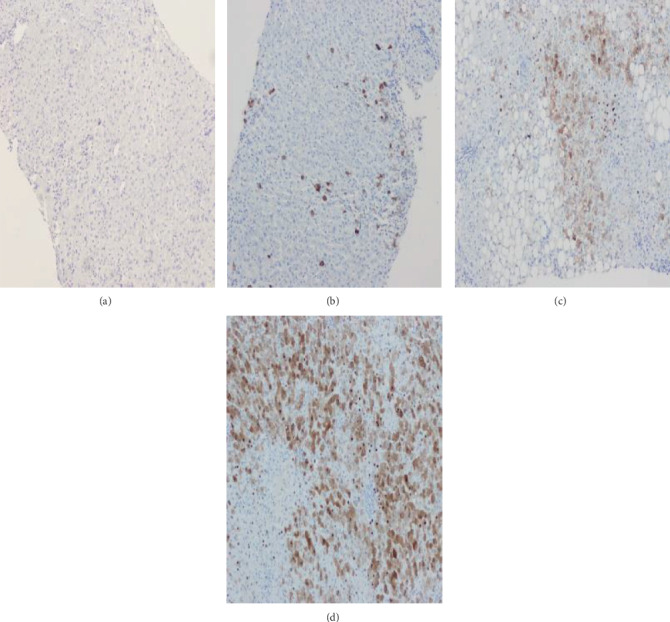
HBcAg expression intensity was classified into four types in this study. (a) The 0 point group showed HBcAg expression in less than 5% of hepatocytes (immunohistochemical stain for HBcAg, ×200). (b) The 1 point group showed HBcAg expression in 5%-33% of hepatocytes (immunohistochemical stain for HBcAg, ×200). (c) The 2 point group showed HBcAg expression in 34%-66% of hepatocytes (immunohistochemical stain for HBcAg, ×200). (d) The 3 point group showed HBcAg expression in over 66% of hepatocytes (immunohistochemical stain for HBcAg, ×200).

HBcAg expression intensity was classified into four types in this study: 0 points, less than 5% hepatitis B core antigen expression; 1 point, 5%-33% hepatitis B core antigen expression; 2 points, 34%-66% hepatitis B core antigen expression; and 3 points, over 66% hepatitis B core antigen expression (Figure 3) [15].

**Figure 4:**
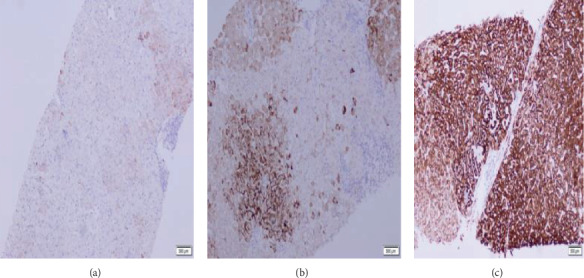
HBsAg expression intensity was classified into three types in this study. (a) The 1 point group showed HBsAg expression in 5%-33% of hepatocytes (immunohistochemical stain for HBsAg, ×200). (b) The 2 point group showed HBsAg expression in 34%-66% of hepatocytes (immunohistochemical stain for HBsAg, ×200). (c) The 3 point group showed HBsAg expression in over 66% of hepatocytes (immunohistochemical stain for HBsAg, ×200).

The HBsAg expression intensity was classified into three types in this study: 1 point, 5%-33% hepatitis B surface antigen expression; 2 points, 34%-66% hepatitis B surface antigen expression; and 3 points, over 66% hepatitis B surface antigen expression (Figure 4) [15].

### 2.3. Antiviral Treatments

All patients were treated with 180 *μ*g iH of PEG-IFN*α*-2a (Pegasys, Roche) once weekly for 48 weeks, and the dosage was adjusted according to any adverse effects identified by their physicians. After initiating the PEG-IFN*α*-2a treatment, the patients were followed-up at the outpatient clinics every 4 weeks. In addition to conventional biochemistry tests, the quantification of serum HBsAg, anti-HBs, HBeAg, anti-HBe, and HBV DNA was performed at baseline and at week 12 or 24, 48, and 72. Complete response (CR) was defined as a serum HBV DNA level less than 500 IU/mL, normal ALT levels (<40 IU/L), and HBeAg seroconversion (HBeAg loss and anti-HBe-positive) at 48 weeks posttreatment and 24 weeks follow-up. Virological response (VR) was defined as a serum HBV DNA level of less than 500 IU/mL. Biochemical response (BR) was defined as ALT and AST levels returning to normal. The following cases were discontinued during treatment: patients who had difficulty tolerating the side effects and patients who refused to continue antiviral therapy. The following cases were discontinued during 24 weeks follow-up: patients who had an elevation of ALT levels >10 times ULN; patients who refused to continue following up and switch to nucleoside analogues (NAs) therapy.

### 2.4. Statistical Analysis

All statistical analyses were performed using SPSS 22.0 software (IBM Co., Armonk, NY, USA). Continuous variables are presented as the mean ± standard deviation (or median and range), and the Mann-Whitney *U* test was used to compare nonparametric continuous variables. Categorical variables are expressed as the frequency and percentage and were analysed with the chi-square test. The correlation between groups and the results was analysed by Spearman's rank correlation analysis. Multivariate logistic regression was used to determine the independent predictors of the relationship between the complete response rate and related factors. A two-sided *P* value < 0.05 was considered statistically significant.

## 3. Results

### 3.1. Baseline Characteristics of Different Degrees of Hepatic Steatosis in CHB Patients

The baseline characteristics are listed in Table 1. The patients were divided into four groups according to their degrees of hepatic steatosis. CHB patients with hepatic steatosis were more likely to be male, be aged >40 years, have a BMI > 23 kg/m^2^, have hyperuricaemia, have higher levels of TC, TG, GLU, and UA, and have lower levels of ALT and AST, as shown in Table 1.

Continuous variables are expressed as the mean ± SD or median (interquartile range), and categorical variables are described by counts and proportions. Pearson's chi-square test and the Student's *t*-test were used for statistical analysis. Abbreviations: TC: total cholesterol; TG: triglycerides; UA: uric acid; GLU: glucose; ALT: alanine aminotransferase; AST: aspartate aminotransferase, HBV: hepatitis B virus; BMI: body mass index.

### 3.2. Correlation between Hepatic Steatosis and Hepatic Histological Necroinflammation, Fibrosis Stage

The baseline hepatic histological necroinflammation and hepatic steatosis stages were negatively correlated. The more severe the hepatic steatosis was, the less severe the liver inflammation activity (Table 2). There was no correlation between the degree of liver fibrosis and hepatic steatosis (Table 3).

The categorical variables are described by counts and proportions. The Pearson chi-square test, Spearman's rank correlation analysis, and coefficient of contingency were used for the statistical analysis.

The categorical variables are described by counts and proportions. The Pearson chi-square test, Spearman's rank correlation analysis, and coefficient of contingency were used for the statistical analysis.

### 3.3. Risk Factors Associated with Significant Histological Abnormalities

Clinical parameters (sex, age, disease course, baseline TC, baseline TG, metabolic syndrome, baseline ALT, baseline AST, baseline HBV DNA, BMI > 23 kg/m^2^, baseline UA, and GLU) were analysed by multivariate regression and were correlated with liver histological abnormalities (Table 4). BMI > 23 kg/m^2^ (*P* < 0.001) was independently associated with the presence of hepatic steatosis in CHB patients. Baseline AST (*P* = 0.029) and baseline UA (*P* = 0.040) were independently associated with significant hepatic steatosis in CHB patients. Baseline AST (*P* = 0.038) was independently associated with significant fibrosis in CHB patients.

### 3.4. Correlation between Hepatic Steatosis and the Rates of VR, BR, and CR after PEG-IFN*α*-2a Treatment

The VR, BR, and CR rates were observed in the four groups after 48 weeks of treatment. The rates of VR, BR, and CR differed significantly between the four groups (*P* < 0.05). The more severe the hepatic steatosis was, the lower the VR, BR, and CR rates (Table 5).

Categorical variables are described by counts and proportions. The Pearson chi-square test was used for the statistical analysis.

### 3.5. Correlation between Hepatic Steatosis and the Rates of VR, BR, and CR after 24 Weeks of Follow-Up

During 24 weeks follow-up, there were thirteen patients who had an elevation of ALT levels >10 times ULN discontinued follow-up and switch to NAs therapy and seven patients intended to NA therapy so drop out continue the follow-up. 206 patients finished the follow-up.

The VR, BR, and CR rates were observed in the four groups after 24 weeks of follow-up. The rates of VR, BR, and CR differed significantly between the four groups (*P* < 0.05). The more severe the hepatic steatosis was, the lower the VR, BR, and CR rates (Table 6).

Categorical variables are described by counts and proportions. The Pearson chi-square test was used for the statistical analysis.

### 3.6. Correlation between Hepatic Steatosis and HBcAg and HBsAg Expression

The four degrees of hepatic steatosis have different HBcAg expression patterns. The rate of HBcAg-negative and c-HBcAg was 43.70% in the S0 group, whereas it was 31.58%, 31.82%, and 33.33% in the S1, S2, and S3 groups, respectively (*P* = 0.015) (Table 7). However, the expression levels of HBsAg and HBcAg did not differ significantly between groups (Tables 8 and 9).

## 4. Discussion

In this study, we compared the baseline clinical data of CHB patients with different degrees of hepatic steatosis. CHB patients with hepatic steatosis were more likely to have higher levels of TC, TG, GLU, UA, and BMI; be of themale sex; be aged >40 years; have hyperuricaemia and a BMI > 23 kg/m^2^; and have significantly lower ALT and AST levels than those without hepatic steatosis. The UA and TG levels and the incidence of hyperuricaemia gradually increased with the degree of hepatic steatosis, whereas the proportion of patients over 40 years old gradually decreased. The combined presence of CHB and hepatic steatosis was associated with metabolic disorders and had caused diabetes mellitus, atherosclerosis, and cardiovascular and cerebrovascular events [16–18]. The proportion of patients aged >40 years gradually decreased with higher degrees of hepatic steatosis, suggesting that lipocytes were necrotic and hepatic fibrous tissue gradually proliferated with age.

The multivariate logistic regression analysis showed that a BMI > 23 kg/m^2^ was an independent risk factor for CHB with hepatic steatosis, which suggests that overweight or obesity is an important feature of CHB patients with hepatic steatosis and has caused or may be related to increased insulin resistance [19, 20]. Insulin resistance can impair the ability of surrounding tissues to absorb and utilize glucose, resulting in the deposition of a large amount of TG in the liver, which leads to the formation of hepatic steatosis. Furthermore, the multivariate logistic regression analysis showed that baseline levels of AST and UA were independent risk factors for CHB with significant hepatic steatosis (hepatic steatosis ≥ S2), and baseline AST levels were independent risk factors for significant liver fibrosis (fibrosis ≥ F2). There was no significant difference in HBV DNA titres between the four groups, which indicates that HBV replication was not associated with the presence of hepatic steatosis.

In this study, we present our findings that a higher degree of hepatic steatosis is associated with a lower level of inflammation in the liver tissue; moreover, there was no correlation between the degree of liver fibrosis and the degree of hepatic steatosis. One domestic study [15] showed that the mechanism by which a higher degree of hepatic steatosis results in lower liver inflammation may be the ability of hepatic steatosis to weaken the expression intensity of HBsAg and HBcAg in liver tissue. Thus, the ability of viral proteins to activate cellular immune responses is reduced, which reduces the destruction of infected hepatocytes and causes slight liver inflammation.

However, our study investigated the difference in expression levels of HBsAg and HBcAg in different groups and found that there was no significant difference in the expression levels of HBsAg and HBcAg between groups with different severities of hepatic steatosis. Furthermore, we demonstrated that HBcAg expression patterns were related to hepatic steatosis. In the group without steatosis, 43.70% of patients were HBcAg-negative and c-HBcAg, whereas 31.77% of patients with steatosis were HBcAg-negative and c-HBcAg. One study [14] showed that the expression patterns of HBcAg-negative and c-HBcAg in the liver tissues of CHB patients usually occurred in the stage of immune clearance and active liver inflammation, whereas the cd-HBcAg group and the m-HBcAg group were often in the stage of immune tolerance and had mild liver inflammation, resulting in a lower antiviral effect. Therefore, the effect of PEG-IFN*α*-2a in CHB patients with hepatic steatosis was poor. Our study compared the rates of VR, BR, and CR among the four degrees of hepatic steatosis after 48 weeks of treatment and 24 weeks of follow-up. There was a significant difference between the four groups. With the increase in the degree of steatosis, the rates of VR, BR, and CR decreased significantly. Therefore, we should take effective measures for the prevention and treatment of hepatic steatosis to improve the efficacy of PEG-IFN*α*-2a.

In conclusion, ALT and AST levels and the inflammatory activity of liver tissue in CHB patients with steatosis were significantly lower than those in patients without steatosis; this result may be due to the low expression of the HBcAg-negative and c-HBcAg type in liver tissue that leads to a poor immune response, eventually causing a poor antiviral effect. Antiviral therapy with PEG-IFN*α*-2a is not appropriate for CHB patients with hepatic steatosis. Conversely, hepatic steatosis is mainly related to sex, baseline TC, TG, UA, GLU, and BMI but is unrelated to HBV DNA load. Therefore, we can improve the clinical efficacy of PEG-IFN*α*-2a in CHB patients with hepatic steatosis by weight control, alcohol abstinence, diet adjustment, and insulin resistance improvement.

There are several limitations of the present study. We evaluated the efficacy of PEG-IFN*α*-2a treatment for 48 weeks and 24 weeks of drug withdrawal without evaluating the sustained efficacy of 48 weeks of drug withdrawal because several patients needed antiviral therapy again due to the significantly elevated serum ALT and AST levels after the drug withdrawal for more than 24 weeks, which would have led to a large dropout rate. Our study was performed in a single centre and lacked sufficient samples, particularly in the severe steatosis group. Therefore, a multicentre, a balanced number of each group, and a large-sample study should be conducted in the future.

## Figures and Tables

**Table 1:** Baseline characteristics of 226 chronic hepatitis B patients.

Variable	Degrees of hepatic steatosis				*F* value	*P* value
	S0 (*N* = 119)	S1 (*N* = 76)	S2 (*N* = 22)	S3 (*N* = 9)		
Male (*n* (%))	82 (68.91%)	65 (85.53%)	21 (95.46%)	9 (100%)	14.828	0.002
Age	29.50 ± 5.47	31.46 ± 8.40	29.41 ± 6.19	27.00 ± 7.38	2.063	0.106
Age >40 (*n* (%))	6 (5.04%)	15 (19.74%)	1 (4.55%)	0 (0%)	13.28	0.004
Course of disease	8.57 ± 5.96	8.17 ± 5.29	8.98 ± 5.84	10.0 ± 9.50	0.327	0.806
TC	4.68 ± 0.80	5.15 ± 0.94	5.05 ± 1.11	5.43 ± 1.25	5.703	0.001
TG	0.56 ± 0.45	1.34 ± 0.68	1.42 ± 0.44	1.76 ± 0.63	13.180	0.001
UA	345.78 ± 69.38	370.10 ± 78.07	381.22 ± 101.58	429.69 ± 65.93	4.271	0.006
Hyperuricaemia (*n* (%))	18 (15.13%)	17 (22.37%)	9 (40.91%)	6 (66.67%)	18.255	0.001
Metabolic syndrome (*n* (%))	1 (0.84%)	3 (3.95%)	1 (4.55%)	1 (11.11%)	4.537	2.063
GLU	4.98 ± 0.50	5.35 ± 0.63	5.21 ± 0.36	5.67 ± 1.05	9.404	0.001
ALT	257.39 ± 175.17	186.14 ± 162.63	182.45 ± 108	137.44 ± 61.88	4.347	0.005
AST	126.72 ± 79.55	88.07 ± 67.82	76.95 ± 39.30	57.44 ± 24.10	7.560	0.001
HBVlog10 (IU/mL) DNA	7.17 ± 0.82	6.96 ± 0.98	7.23 ± 1.15	7.47 ± 0.69	1.224	0.302
HBsAg (log IU/mL)	4.12 ± 1.14	4.04 ± 0.91	4.23 ± 1.18	4.19 ± 1.13	1.273	0.283
HBV genotype					1.00	0.616
B	35 (29.41%)	23 (30.26%)	7 (31.83%)	3 (33.33%)		
C	78(65.55%)	50 (65.79%)	14(63.64%)	6 (66.67%)		
Mix	6(5.04%)	3 (3.95%)	1 (4.55%)	0		
BMI	20.87 ± 1.96	24.98 ± 3.82	24.91 ± 3.91	25.78 ± 3.44	36.996	0.001
BMI > 23 kg/m^2^	14 (11.76%)	54 (71.05%)	14 (63.64%)	7 (77.77%)	80.932	0.001

**Table 2:** The correlation between baseline hepatic histological necroinflammation and baseline hepatic steatosis in each group.

Feature	Total cases	G1	G2	≥G3	Spearman's rank correlation analysis	Coefficient of contingency
S0	119	2	74	43	0.036	-0.140
S1	76	1	55	20		
S2	22	0	16	6		
S3	9	0	9	0		

**Table 3:** The correlation between baseline hepatic fibrosis stage and baseline hepatic steatosis degree.

Feature	Total cases	F1	F2	≥F3	Spearman's rank correlation analysis	Coefficient of contingency
S0	119	69	40	10	0.202	-0.085
S1	76	50	20	6		
S2	22	2	6	14		
S3	9	7	2	0		

**Table 4:** Multivariate analysis of clinical parameters independently associated with significant histological abnormalities.

Patient characteristics	Clinical parameters	OR (95% CI)	*P* value
Presence of hepatic steatosis	BMI > 23 kg/m^2^	5.481 (2.992-10.041)	0.001
Presence of significant hepatic steatosis (≥S2)	Baseline AST	0.978 (0.959-0.998)	0.029
	Baseline UA	1.006 (1.000-1.012)	0.040
Presence of significant fibrosis (≥F2)	Baseline AST	1.006 (1.000-1.012)	0.038

**Table 5:** The virological response, biochemical response, and complete response in the four groups after 48 weeks of treatment.

Groups	Cases	The rate of VR	The rate of BR	The rate of CR
CHB with S0	119	42.9%	75.6%	28.6%
CHB with S1	76	30.3%	40.9%	14.5%
CHB with S2	22	18.2%	31.8%	9.1%
CHB with S3	9	0%	22.2%	0%
*F* value		11.777	32.228	9.552
*P* value		0.007	0.001	0.018

**Table 6:** The virological response, biochemical response, and complete response in the four groups after 24 weeks of follow-up.

Groups	Cases	The rate of VR	The rate of BR	The rate of CR
CHB with S0	110	40.0%	68.2%	34.5%
CHB with S1	72	26.3%	36.1%	20.8%
CHB with S2	18	16.7%	27.8%	11.1%
CHB with S3	6	0%	16.7%	0%
*F* value		8.505	25.988	8.390
*P* value		0.029	0.001	0.032

**Table 7:** The difference in HBcAg expression patterns in different degrees of steatosis.

Degrees of steatosis	Total cases	HBcAg-negative group	c-HBcAg group	cd-HBcAg group	m-HBcAg group	Pearson's chi-square test
S0	119	5	47	63	4	0.015
S1	76	10	14	48	4	
S2	22	3	4	13	2	
S3	9	0	3	5	1	

**Table 8:** The difference in HBsAg expression levels in different degrees of steatosis.

Degrees of steatosis	Total cases	1	2	3	Rank test
S0	119	8	10	101	0.085
S1	76	3	13	60	
S2	22	1	5	16	
S3	9	0	1	8	

**Table 9:** The difference in HBcAg expression levels in different degrees of steatosis.

Degrees of steatosis	Total cases	0	1	2	3	Rank test
S0	119	8	28	20	63	0.257
S1	76	10	13	17	36	
S2	22	3	4	5	10	
S3	9	0	1	3	5	

## Data Availability

The data used to support the findings of this study are included within the article.
